# Multistable Decision Switches for Flexible Control of Epigenetic Differentiation

**DOI:** 10.1371/journal.pcbi.1000235

**Published:** 2008-11-28

**Authors:** Raúl Guantes, Juan F. Poyatos

**Affiliations:** 1 Department of Condensed Matter Physics, Facultad de Ciencias C-XVI, Universidad Autónoma de Madrid, Madrid, Spain; 2 Instituto Nicolás Cabrera, Facultad de Ciencias C-XVI, Universidad Autónoma de Madrid, Madrid, Spain; 3 Logic of Genomic Systems Laboratory, Spanish National Biotechnology Centre, Consejo Superior de Investigaciones Cientificas, Madrid, Spain; University of Tokyo, Japan

## Abstract

It is now recognized that molecular circuits with positive feedback can induce two different gene expression states (bistability) under the very same cellular conditions. Whether, and how, cells make use of the coexistence of a larger number of stable states (multistability) is however largely unknown. Here, we first examine how autoregulation, a common attribute of genetic master regulators, facilitates multistability in two-component circuits. A systematic exploration of these modules' parameter space reveals two classes of molecular switches, involving transitions in bistable (progression switches) or multistable (decision switches) regimes. We demonstrate the potential of decision switches for multifaceted stimulus processing, including strength, duration, and flexible discrimination. These tasks enhance response specificity, help to store short-term memories of recent signaling events, stabilize transient gene expression, and enable stochastic fate commitment. The relevance of these circuits is further supported by biological data, because we find them in numerous developmental scenarios. Indeed, many of the presented information-processing features of decision switches could ultimately demonstrate a more flexible control of epigenetic differentiation.

## Author Summary

An essential attribute of living cells is the capacity to select among various alternatives when confronted with external or internal cues. These decisions can be directly linked to survival, as the sporulation/competence choice in the bacterium *Bacillus subtilis*, or be involved in the establishment of developmental programs from pluripotent stem cells. How are these decisions controlled at the molecular level? Recent studies have identified the presence of a few master regulator genes whose activity is crucial to drive cells to a particular fate. These genes usually exhibit autoregulation and appear in combination with a second molecular partner constituting a minimal regulatory circuit. Here, we investigate how such two-component architectures endow cells with more than two epigenetic states. This ability not only enhances the number of potential developmental outcomes, in a given context, but also drastically increases cell signal processing. We show how these control modules can implement a number of complex computational tasks such as discrimination of stimulus amplitude, duration, or relative timing. Similar aspects have been discussed in relation to neural dynamics in cortical circuits, which suggests the use of equivalent computational strategies and circuit design in the control of biological decision-making.

## Introduction

The capability of cells to present different stable expression states while maintaining identical genetic content plays a significant role in differentiation, signal transduction and molecular decision-making. These epigenetic phenotypes are partly associated to changes in genomic structural features, including several types of chromatin and DNA modifications [1]. Alternatively, it is also believed that in some cases they are induced by the action of underlying genetic regulatory circuits, exhibiting a positive feedback loop configuration. Recent studies have experimentally confirmed this latter prediction, both in natural and synthetic systems, e.g., [2],[3], which originated back in the early days of microbial molecular genetics [4] and systems theory [5].

A positive feedback topology is nevertheless not sufficient to generate distinct epigenetic states. In addition, the circuit should display some degree of nonlinearity, i.e., sigmoidality, on its constituent interactions [6]–[8]. This sigmoidal behavior is typical of many molecular interactions and endows these genetic modules, now interpreted as dynamical systems, with multistability, i.e., the possibility to find the system in alternative steady states under conditions in which all its biochemical parameters are fixed. These equilibria define the different stable expression states regulated by means of the molecular loop.

How does the particular structure of a given positive feedback influence its function? Considering multistability as the most prominent attribute of these architectures, one could argue that genetic design does not really matter, as soon as sigmoidal interactions are achieved in some effective way. Careful analysis of some of the recent experimental reports seems to indicate the contrary. Two general patterns can be suggested. First, positive feedback loops at the core of more complex regulatory networks generally consists of simple structures controlling cell fate decisions. This is normally associated to two complementary expression states, i.e., bistability (like the p42–Cdc2 system involved in *Xenopus* oocytes maturation [9], or the bacteriophage λ genetic switch [10]), but three states is also been recently discussed [11],[12]. In comparison, loops relevant to signal transduction, or more broadly to conditions where complex biochemical information-processing is required, are commonly constituted by many components [13]–[15]. These architectures can even regulate the plain presence of mono or bistability, e.g., as a function of the time the activating stimulus is applied [16].

The proposed patterns lead to a set of interesting questions. When is multistability, understood as at least three possible expression states, relevant in differentiation as opposed to bistability? Which simple feedback loop architectures can produce it, and what biologically relevant parameters do we need to quantify in order to predict these behaviors? Moreover, we can also ask to what extent complex feedback topologies are necessarily required for multifaceted information-processing and for the execution of elaborated developmental programs.

**Figure 1:**
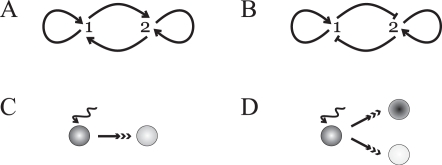
Circuit topologies and canonical differentiation types. Mutual-activation (A) or mutual-inhibition (B) circuits constituted by two master regulators (denoted 1 or 2). Both modules exhibit autoregulation of their components. (C,D) Differentiation as a progression or decision. In (C) a cell changes its expression state to a new one as a result, for instance, of a signalling event (wiggle arrow). This is implemented by a progression switch only requiring two expression states. Alternatively, a decision switch can drive the initial expression state to two different ones and thus needs three expression states, corresponding to the states before and after the decision. Different cell colors correspond to distinct expression states. See main text for details.

To address some of these issues, we first investigate the number of available expression states of two minimal complementary systems—a two-component mutual-activation and mutual-inhibition circuits—whose constituents are autoregulated (Figure 1A and 1B). Autoregulation is a pervading feature of many eukaryotic master regulators [17],[18], a property usually believed to merely impart stability to the associated regulations [18]–[20]. In this context, we show how it determines, in competition with the crossregulatory interactions, the ranges of mono, bi and multistability. Second, we discuss how these topologies facilitate two main switch classes involving transition between expression states in bistable and multistable regimes. We term these classes progression and decision switches, respectively. While multistable decision switches have been recently suggested as a rationale to explain co-expression of antagonistic master regulators in lineage specification scenarios [21],[22], we show that these switches not only appear in mutual-inhibition architectures. More importantly, we analyze several complex information-processing tasks in decision switches, such as signal strength [23] and duration [24] discrimination, stochastic fate control [25],[26] and flexible discrimination [27],[28]. This demonstrates that simple architectures can indeed show rich computations, an ability related to the presence of multistability and thus to autoregulation. We revisit this relation next to highlight how autoregulation compensates or amplifies transient expression differences between circuit components to induce robust coexisting stable expression states. We close by discussing the implications of these findings, and the specific architectures considered, in various cellular contexts.

## Results/Discussion

### Models

To describe dynamical aspects of genetic regulatory networks one can generally adopt two contrasting strategies. The first one is to collect all available molecular information about the regulatory interactions of a specific system. This allows to present the putative regulatory network involved in the mechanism under study, which can then be quantitatively described by using a particular mathematical formalism, e.g., a set of ordinary differential equations. While this method can be helpful to describe the dynamics of a very specific system, it usually incorporates a degree of complexity that can sometimes hide the key dynamical aspects, and molecular players, determining network behavior (with the additional drawback that new molecular agents could always be discovered and thus modify both network topology and dynamics). An alternative approach is to propose simplified mathematical models based on a number of realistic assumptions. Simple models help in the identification of basic design principles, might act as effective descriptions of more complex circuits and, as we see below, can actually correspond to extant regulatory modules found in several biological scenarios. These models also circumvent the lack of quantitative molecular details required in the more specific studies. We follow here this second approach.

We thus introduce a two-component mathematical model to analyze the dynamical behavior of the mutual-activation/mutual-inhibition topologies (see equations in Materials and Methods, Figure S1, Figure S2, and also Text S1 for further details). In this model, autoregulatory and crossregulatory interactions between components were represented by Hill equations. This is a widely used approximation as molecular interactions are usually known to behave in a sigmoidal fashion [6]. Indeed, similar simplified models have been used to describe the coexistence of several expression states in specific cell fate systems, such as those involved in hematopoiesis [11],[12],[22] or embryonic stem cell differentiation [29]). In our case, we present this model as part of a general framework in relation to a broad number of biological scenarios (see Table 1), and fully characterize the type of information-processing features these circuits exhibit and their potential significance for a more flexible control of epigenetic differentiation.

**Table 1:** Genetic circuitry in eukaryotic differentiation.

	System	Components	Mediators	Interaction Type	Output Fates
Mutual activation topology	Embryonic stem cells [36],[39]	(Oct4/Sox2,Nanog)	Direct [58]	Transcriptional	(low,low)→differentiation
					(high,high)→self-renewal
	Neurogenic network [59]	(Ac,Sc)	Da	Transcriptional	(low,low)→epidermal
					(high,high)→neural
	Myogenic differentiation [17]	(MyoG,Mef2C)	myogenic bHLH factors	Transcriptional	(low,low)→precursor cells
					(high,high)→muscle cells
	Pancreatic development [54]	(Sox9,FoxA2)	Direct [54],[60]	Transcriptional	(low,low)→self-renewal
					(high,high)→endocrine diff.
	*Xenopus* oocyte maturation [9]	(p42,Cdc2)	Mos, Myt1	Post-translational	(low,low)→immature oocyte
					(high,high)→mature oocyte
	Apoptosis [61]	(Casp3, Casp9)	Direct	Post-translational	(low,low)→cell survival
					(high,high)→apoptosis
Mutual inhibition topology	Mammalian embryogenesis [37]	(Cdx2,Oct3/4)	Direct	Transcriptional cooperative	(high,high)→precursor cells
					(high,low)→trophectoderm
					(low,high)→inner cell mass
	Hematopoietic development [12],[41]	(GATA1,PU.1)	Direct	Transcriptional cooperative	(low,low)→priming state
					(high,low)→erythroid/megakaryocytic
					(low,high)→myelomonocytic
	T-cell differentiation [62]	(T-bet,Gata3)	ITK	Post-translational	(high,high)→pluripotent state
					(high,low)→Th-1 cells
					(low,high)→Th-2 cells
	Visual system specification [63]	(Pax6,Pax2)	Direct	Transcriptional	(high,high)→early eye epithelium
					(high,low)→Optic cup
					(low,high)→Optic stalk
	*Drosophila* eye development [26]	(Wts,Melt)	Unknown	Transcriptional	(high,low)→‘Yellow’ photoreceptor
					(low,high)→‘Pale’ photoreceptor
	*C. elegans* gustatory neurons [64]	(*die–1*,*cog–1*)	miRNAs	Transcriptional	(high,high)→equipotent
					(high,low)→ASEL neuron
					(low,high)→ASER neuron

### Biological Determinants and Circuit Control of Multistability

What specific biological features determine multistability? We address this question by identifying a minimal set of biological determinants able to characterize circuit behavior. This analysis also helps us to highlight some unexpected features of the relation between module structure and epigenetics, and to introduce two main types of switches associated to the circuit dynamics.

#### Phenotypic map

The epigenetic profile exhibited by a particular positive feedback topology depends on the activation (inactivation) of the expression of its molecular constituents. This expression pattern is determined by the number of available equilibrium states of the system, which in turn depends on the specific values of its parameters. Thus, by changing the parameters, we can ultimately predict all the potential epigenetic regimes that a circuit can present. Such parameter space, commonly used in the study of dynamical systems [30], acts in this context as a truly phenotypic map, since it fully predicts the circuit potential behavior and can also guide its experimental characterization [2],[3],[11],[31]. What defines the phenotypic maps of the mutual-regulation topologies? Three main biologically relevant parameters influence their structure (see also Materials and methods): the relative strength between auto (*ρ*) and crossregulatory (*ν*) links, the ratio between the thresholds of activation of these two link types (*σ*), and the magnitude of the basal production rate (*a*). Note also that regions within these maps could only display areas with one to four possible epigenetic states—(low,low), (low,high), (high,low) and (high,high), with low/high denoting expression levels of the corresponding gene—as these are two-component systems.

**Figure 2:**
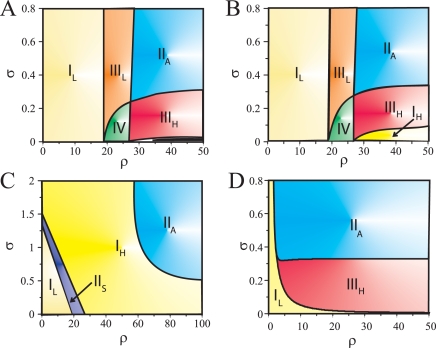
Phenotypic map for mutual-inhibition and mutual-activation circuits. This map shows the areas of coexistence of several expression states (multistability) in a *σ*-*ρ* parameter space (*a* = 0.1, A–C). These regions are: I_{L,H}_; one (low,low)/(high,high) expression state, II_{S,A}_; coexistence of (low,low)–(high,high), symmetric, and (low,high)–(high,low), antisymmetric, expression states, III_{L,H}_; tristability with two antisymmetric states and one symmetric state, low or high, IV; coexistence of four expression states. The phenotypic map for mutual-inhibition (A, *ν* = 0) is structurally similar to that of weak mutual-activation (B, *ν* = 2). Multistability generally arises when increasing autoactivation, *ρ*, while balancing the threshold of activation between the auto and crossregulation. Strong crossactivation (C), *ν* = 20, enables only mono or bistability. Note also that an increase in basal rates (D, *a* = 1 with *ν* = 0) reduces the presence of low expression states.

We show several phenotypic maps in Figure 2. In a first glimpse, we see different regions associated to the coexistence of distinct expression patterns. Strong autoactivation appears here as a necessary condition for the existence of more than one single expression state (*ρ*≃18, Figure 2A and 2B). Beyond this threshold two broad regimes are found, depending on which regulatory interaction is dominant. In a regime where the autoregulation is more likely than crossregulation (*σ*≪1), a symmetric state with high levels of expression is available (IV and III_H_, Figure 2A and 2B). This possibility is lost when crossregulation becomes more active (III_L_ and II_A_, Figure 2A and 2B). We can also see how the low-expression symmetric state disappears as autoactivation strength increases (IV→III_H_ and III_L_→II_A_, respectively). We further observe a qualitatively similar phenotypic map for both mutual inhibition (Figure 2A, *ν* = 0) and weak mutual activation (Figure 2B, *ν* = 2). The map is thus structurally stable for small changes in *ν*, even though they represent fundamentally different circuits.

The previous stereotypic behavior changes for strong crossactivation (*ν*≫1, Figure 2C). In this case, coexistence of more than two expression states is not possible. For *ρ*≈*ν*, we see the expected map of a simple switch based on mutual activation without autoregulation. This is characterized by the existence of two symmetric expression states—(low,low) and (high,high)—with a bistability region (II_S_). A different behavior arises when autoactivation strength dominates crossactivation, *ρ*≫*ν*. In this situation, two anti-symmetric states are available for *σ*≈1, i.e., when auto and crossinteraction thresholds are comparable. This is a somehow unexpected effect, since one would not anticipate that a module with high strength of both types of interactions could produce as stable expression levels two anti-symmetric states. Finally, an increase in basal rates (*a*), the third relevant parameter of the system, normally unstabilizes low-expression states. This implies shrinkage, or total disappearance, of regimes IV and III_L_ in Figure 2A and 2B, as autoactivation would additionally enhance such high basal production (Figure 2D and Figure S3A and S3B).

#### Progression and decision switches

How can the epigenetic state of these circuits be modified? A change of gene expression is induced by external factors, e.g., due to a signalling event, which effectively modify the parameters of the circuits, and thus their location on the phenotypic map. This can generally happen in two ways. In some situations, the initial expression level progresses to a new one, this being the only possible steady state of the system (Figure 1C). For instance, a transition from a state in which both genes are highly expressed to one in which they can only be weakly expressed, e.g., II_S_→I_L_ in Figure 2C (by means of a change in autoactivation strength, *ρ*). Alternatively, the change of gene expression can be understood as a decision, in which the initial expression state could evolve towards several alternative ones (Figure 1D). This could happen when a circuit in a symmetric high expression state changes to either one of the two asymmetric ones (for instance, a III_H_→II_A_ transition in Figure 2A). Combinations between these canonical types of binary choices are likely at the core of more complex scenarios [12],[21].

Accordingly, we suggest that molecular switches driving these transitions can be classified as progression or decision switches. A progression switch drives the system to a final monostable epigenetic regime, while a decision switch takes the circuit towards a bi- or multistable regime. The phenotypic map shows then different parameter areas between which a particular topology could enable these switches.

**Figure 3:**
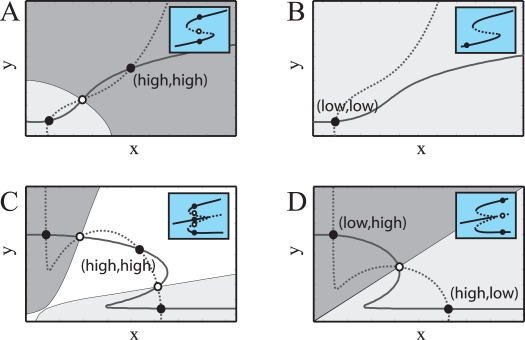
Switch classes as changes (bifurcations) in gene expression. The intersection between the circuit response curves or nullclines (lines in the *x*-*y* planes) identifies the system steady states, these being either stable (filled circles), or unstable (empty circles). In this way, a range of different initial concentrations of the circuit components (basin of attraction; white and light/dark gray areas) ends up in the same expression state. A progression switch is associated to a transition from a high (A) to a low (B) expression state, crossing a bistable regime. This is a saddle-node bifurcation, insets (A,B), where the magnitude and types of available equilibria are plotted as a given parameter changes in the *x*-axis (solid line; steady state, dotted line; unstable state). A decision switch corresponds however to a transition in which the initial symmetric expression state (high,high) becomes unstable (C), and thus only two expression states, (high,low) and (low,high), remain (D). This transition could proceed in a all-or-none (C,D) or graded fashion (Figure S4) and is linked to a pitchfork bifurcation, insets (C,D). Note that in this case the circuit goes from having three to two coexisting expression states.

It is also important to distinguish how the state previous to the expression change is abandoned. In a progression switch, this equilibrium is no longer available after a given stimulus strength, and the system necessarily jumps toward a new expression state. In comparison, the initial state of a decision switch does not disappear but becomes unstable. While both situations seem equivalent experimentally, their implications for switch function are totally different. The unstable state acts effectively as a boundary splitting mutually exclusive domains of expression (see below and also next section). This qualitative reasoning can be formally described using the language of Dynamical Systems Theory, where the previous transitions correspond to steady state bifurcations [30]. Thus, a progression switch corresponds to a saddle-node bifurcation and a decision switch to a pitchfork bifurcation (insets Figure 3). We illustrate this by plotting the response curves or nullclines (solving Equation 1 in Materials and Methods for 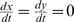), whose intersections identify the steady states of the system, and their basin of attraction.

In Figure 3A, a cell population exhibiting transient states of the circuit components (*x*, *y*) whose levels of expression were scattered through the dark grey basin converge after some time to the (high,high) steady state. The presence of an inhibitory signalling event makes this state disappear by means of a saddle-node bifurcation (insets in Figure 3A and 3B). The whole population abruptly switches now to the (low,low) state (Figure 3B, note that there is just a single basin of attraction in this regime). Analogously, transient expression levels within the white domain of Figure 3C. evolve to the (high,high) state. However, the presence of an inhibitory signal in this case unstabilizes, rather than destroys, this state through a pitchfork bifurcation (inset Figure 3C and 3D). The population then is divided in two different ones by expressing two exclusive states—(high,low) or (low,high) in Figure 3D. This is due to the fact that the initial distribution falls in the two different basins of attraction in the new situation (dark grey and light grey in Figure 3D). This process acts as an example of a decision which is in this case irreversible and all-or-none (subcritical pitchfork, inset Figure 3C and 3D) but that could also be reversible and graded (supercritical pitchfork, Figure S4).

### Decision Switches as Rich Signal-Processing Units

What type of signal processing enables the presented switches? In the following, we show the potential of decision switches to robustly discriminate several characteristics of biochemical stimuli, e.g., strength, duration, timing, etc. These capacities offer flexible control of epigenetic expression, far beyond that attributed to bistable (progression) switches.

#### Differential signal processing

We first imagine a situation in which a decision switch, initially in a symmetric state of high expression—(*x*, *y*) = (high,high)—is subjected to a transient signal pulse. This pulse acts on both components by increasing their degradation rate with the very same strength, as it could be the case, for instance, when some sort of post-translational modification effectively inactivates the corresponding proteins. While the signal is affecting the two components for the same amount of time, the module remains in the symmetric expression state. A situation that simply reflects the pre-existing balance between crossinteractions, which is not changed by the (symmetric) pulse. However, as soon as there exists some disparity in duration, this leads to the dominance of one of the crossinteractions and to the disappearance of the symmetric steady state. The circuit is driven for this reason to one of the asymmetric expression states. Decision switches can in this way discriminate differences in signal duration and encode this computation in the state expressed after the signaling event.

**Figure 4:**
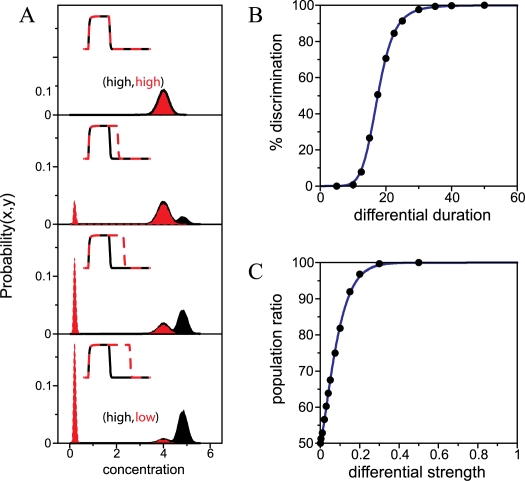
Signal duration and strength discrimination. (A) A signal pulse inducing degradation of *x* and *y* species (insets) is acting with the same strength but different duration in both circuit components (solid and dotted lines for *x*/*y*, respectively). The initial population of cells, all in the symmetric (high,high) expression state (*x*/*y*-component in black/red, respectively), is allowed to evolve after signal removal to the steady state. Increasing differential duration populates the asymmetric (high,low) expression state—the opposite difference in duration would populate the (low,high) state. Circuit parameters are *ρ* = 5, *ν* = 0, *σ* = 0.2, *a* = 1. (B) The fraction of cells going to the correct asymmetric equilibrium is plotted as a function of differential signal duration (filled circles), with a fit to a sigmoidal function (exponent 3.5, threshold 17.5). (C) Strength discrimination: An initial population of cells in the (high,high) state is transiently stimulated with a stronger degradation pulse than (A), but with the same duration in both components (*t* = 50 in adimensional units). With this stimulus, the symmetric equilibrium state becomes unstable and cells compete for the asymmetric (low,high) and (high,low) states. Note that for the same signal strengths, the population is stochastically divided 50% between both attractors, while for increasing differential amplitudes the population is biased towards one of the asymmetric states (filled circles). Solid line in Figure 5C is a fit to a Weibull function. Notably, this fit denotes a similar discrimination performance to that found in cortical circuits [28].

How reliable is this discrimination? For instance, how big should the duration difference be to correctly recognize this distinction? We quantify this ability in Figure 4A and 4B, where we particularly studied a mutual-inhibition decision switch (*ν* = 0). The circuit is initially again in the symmetric high expression state. Because of molecular noise (consequence of the inherent stochasticity of biomolecular reactions [32], see Text S1), this state fluctuates in time around its mean (deterministic) value. This variability could be alternatively understood as the distribution of steady state values that would exhibit a cell population expressing this very same state at a given fixed time (Figure 4A, top). We then consider a stimulus pulse acting on the *y*-element of the module for a longer time (insets Figure 4A). After the pulse, the switch could be driven to the (high,low) expression state, due to the time lapse in which only the *y* protein experiences a larger degradation rate, which in turn weakens its repression on *x*. This epigenetic change depends both on the characteristic time scales of the module response and the magnitude of duration difference between pulses. Thus, if the pulses showed difference in duration and the system is driven to the (high,low) state, we say that it correctly discriminated signal differences.

Figure 4A shows four instances of population distribution after this processing, for increasing differential duration. We find that the larger the difference the bigger the number of cells expressing the expected target state (Figure 4A, top to bottom). A more quantitative analysis reveals that correct discrimination, measured as the percentage of cells in the (high,low) expression state, presents a threshold performance following a sigmoidal curve (Figure 4B). This behavior is associated to the two different time scales determining switch response. On the one hand, differential duration induces a large asymmetry in the space of variables which causes the (high,high) stable state to disappear in a saddle-node bifurcation. The remnant of this stable state produces a slowdown of the dynamics close to this point (a ghost state [30]), the switch exhibits a finite response, and a threshold in discrimination performance arises. This threshold could be biologically important, as it effectively works as a fail-safe mechanism to filter out small signal fluctuations in signal duration. On the other hand, the slope (Hill coefficient) of the sigmoidal response is determined by the switch characteristic dynamics. For the same threshold, a system with larger autoregulation will produce a steeper (all-or-none) behavior in discrimination performance. This is illustrated in Figure S5, where two switches differing only in autoregulation strength are compared.

#### Stochastic fate determination

Discrimination of differences in stimulus strength rather than duration works in a similar vein as before, but it additionally displays other features. A particularly interesting one is linked to the phenomenon of stochastic fate commitment. Cells appear in some occasions to choose among two different phenotypes (cell classes) in a random manner, e.g., [25],[26]. An open question in this context is how such stochastic decision-making leads though to a fixed proportion of classes in a given population, e.g., fixed 20∶80 ratio of competent to noncompetent cells in *B. subtilis* ([33] for a recent review). We discuss here how genetic decision circuits provide a feedback-based mechanism to drive stochastic fate decisions and, in the same way, determine fixed phenotypic proportions. This is achieved as response to signals affecting both circuit components for approximately the same time, but differences in signal strength. This could be also produced by signals with similar strength by operating on circuits with asymmetric properties, e.g., kinetic parameters. Such asymmetry in the kinetic parameters modifies the basin of attraction of the available steady states and ultimately determines the final distribution of the population.

To examine this, we start as in the previous section with a population of cells in the symmetric high expression state, i.e., we consider noisy gene expression. The signal is acting in both components but this time with the very same duration. As a result of this stimulus the circuit remains in the symmetric state, as with duration discrimination, while signal strength is below a particular value. However, when this value crosses a threshold, the symmetric states becomes unstable (insets Figure 3C and 3D). The circuit is then randomly driven to either one of the asymmetric states. The consequence of this fair choice is a final population divided in a 50% ratio in terms of expression. The important issue here is that this balance, and thus the proportion of cell classes, can be modulated by differences of signal strength (Figure 4C). In this sense, the ratio of expression states in the population is encoding the output of the strength discrimination task.

Several features of this process are of interest. First note that, as compared with differential duration, it lacks any threshold. Why is this? Examination of the way the symmetric state is abandoned provides the answer. For strength discrimination this state does not disappear, as in the previous section, but becomes unstable. This distinction is relevant from a biological point of view, although it might not seem to be so *a priori* by simply looking at the equilibrium distributions after the computation. Indeed, both behaviors are quite different from a dynamical perspective since response times in the latter case are much faster. This can be seen by contrasting the discrimination curves (Figure 4C as compared with Figure 4B), where one can see that small differences in signal strength may already produce a large bias in phenotypic proportions. Second, the performance of the strength discrimination follows a Weibull, or stretched exponential, function (Figure 4C). This fit is equivalent to that found in cortical circuits in monkeys [28], where coherence of visual stimulus is discriminated. Reduced neural network models based on known neurophysiology have been proposed to explain such decisions. Interestingly, these models present a mutual inhibition and recurrent self-excitation structure. This similarity emphasizes the presence of equivalent dynamical principles in biological circuits underlying seemingly unrelated functions [34].

#### Influence of signal noise and dynamics

In the examples above, we considered deterministic pulses acting post-translationally (fast time scale). However, realistic signaling events fluctuate in several ways and may also act in slower scales, e.g., by transcriptional regulation. We now briefly study these aspects in connection with stimulus strength and duration computations.

**Figure 5:**
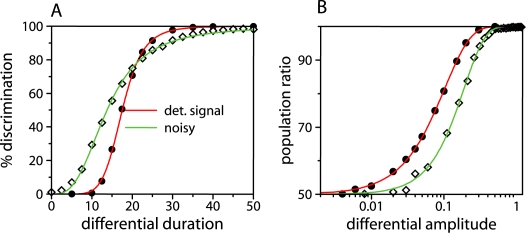
Discrimination performance of a decision switch to differential stimulus duration (A) and amplitude (B). The switch parameters are the same as those in Figure 4, but signal dynamics is now stochastic with fluctuations independent from the circuit components (see Text S1). Signal noise affects duration and amplitude discrimination in different ways.

Signal fluctuations originate opposite effects when considering duration and strength detection. To dissect the role of this noise, we consider again a post-translational (fast) signal and introduce stimulus stochastic dynamics as a birth-death process (see Text S1). In Figure 5A, we compare differential duration discrimination tasks with and without stimulus noise (following poissonian statistics). We find that fluctuations greatly enhance this processing when considering small difference in duration, while it only slightly deteriorate this task in the case of large differences. This noise-induced enhancement is caused by the fact that signal fluctuations help the circuit to abandon the previous ghost state with the corresponding change of gene expression.

What about the discrimination of signal strength? Signal noise modifies this task (stochastic fate determination) in a completely opposite way. In this case, we obtain that correct discrimination gets worse with the presence of stimulus fluctuations (Figure 5B), as noise could drive the system to the complementary asymmetric expression state. These tasks could be also influenced by the signal intrinsic time scale. Slow signals generally require larger differences in stimulus to be properly discriminated, but discrimination performance is less influenced by signal noise (Figure S6).

#### Flexible discrimination

Cells need to discriminate not only relatively simultaneous stimuli, as discussed previously, but also stimuli received with certain time delays. This task is generally linked to the cellular capacity to maintain a memory of recent signaling events, i.e., bistability and positive feedback regulation [10],[13], that could influence future responses. Positive loops can also process delayed signals by inducing a different type of history-dependent dynamics when interlinked with negative feedbacks [16]. In this case, the response of the circuit, gradual or all-or-none, is modulated by the readout of a previous stimulus. We propose now a third scenario, in which a decision switch is able to discriminate delayed signals based on the capability of these circuits to store short-term memories.

**Figure 6:**
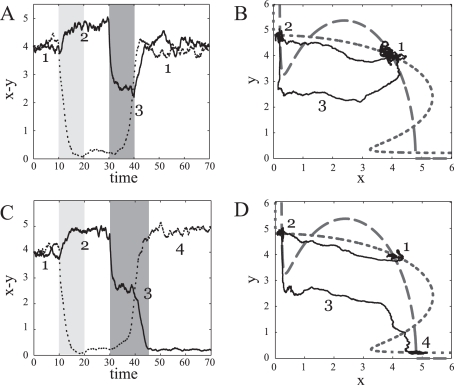
Flexible discrimination. Stochastic time evolution (A,C) of the concentration of the *x* (dotted) and *y* (solid) circuit components implementing flexible discrimination. (B,D) shows the same dynamics in the phase plane, where dashed and dotted lines are the system nullclines (*ρ* = 50, *ν* = 0, *σ* = 0.2, *a* = 0.1). The decision switch, initially in the symmetric expression state, is subjected to two delayed stimuli (gray bars). The first signal, a pulse increasing the degradation rate of the *x* species, takes the system to the (low,high) asymmetric state. This state acts as a short-term working memory. This memory permits the system to discriminate the duration of the next pulse, now acting on *y*, with respect to the previous one. When the second pulse is similar to the first one, the circuit “erases” its memory. Trajectory 1-2-3-1 in (A,B). A larger second pulse drives the system to the (high,low) state; 1-2-3-4 trajectories. Short-term memories enhances thus the reliability of the (high,high)→(high,low) decision and provides a feedback-based mechanism to the dynamics of transient expression states.

We consider two signal pulses separated by a particular time delay, which are operating alternatively on the *x* and *y* components of a decision switch (Figure 6, pulses modify degradation as previously). The first pulse takes the system from an initial symmetric expression state to a new asymmetric state (low,high). When a new stimulus is received, the previous asymmetric state can represent an effective short-term memory linked to the cell's signaling history. This is reflected on how the switch responds to the second signal. When the duration of this signal is similar than the previous one, the circuit goes back to the precursor initial state (Figure 6A and 6B, where we plot time and phase plane evolution), i.e, the memory of the stimulus is erased. A longer pulse would take the system to the opposite asymmetric state (Figure 6C and 6D). The switch thus discriminates the duration of two signals acting on successive times. Such flexible discrimination resembles the one found in cortical circuits, where intermediate stable states serve as a working memory to compare the magnitude of sequential stimuli [27].

### Autoregulation and Multistability

The preceding section discussed several new computational features associated to the presence of multistatibility in these systems. We also argued before how multistatibility is linked to autoregulation, a connection that we now further elaborate. Autoregulation favors multistability by either amplifying or compensating transient differences in expression between the circuit constituents. This modifies the type of steady states usually found in two-component switches; (low,low) or (high,high) expression for mutual-activation and (low,high) or (high,low) expression for mutual-inhibition, but see also [35]. Specifically, amplification habilitates activation switches with asymmetric expression states, while compensation induces the presence of a third symmetric equilibrium state in inhibition switches. This dual role requires autoregulation to be strong enough and dominant over crossregulation (*ρ*>*ν*, *σ*<1) (Figure S7).

**Figure 7:**
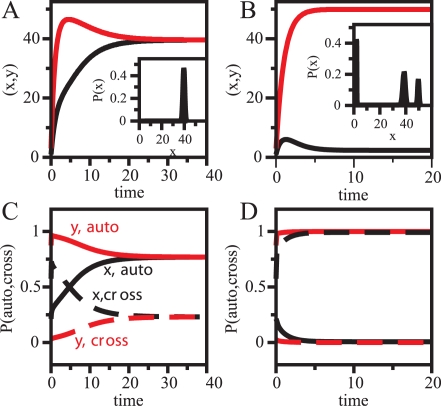
Autoregulation-based amplification. Deterministic trajectories (A,B) and time evolution of different promoter occupancies (C,D) for a mutual-activation switch with strong autoregulation (*ρ* = 50) and intermediate crossinteraction threshold (*σ* = 0.3, basal production *a* = 1). Black lines correspond to the time evolution for the *x* species, and red lines for *y* species. Initial asymmetric conditions are *x*_0_ = 1, *y*_0_ = 3 (non-dimensional units). Insets correspond to a population distribution for the *x* species if stochastic gene expression is considered (Text S1). (A,C) Moderate mutual interaction strength (*ν* = 5). Only a symmetric high expression state exists (see inset). In (C), solid lines correspond to probabilities of auto occupation of the promoter sites, while dashed lines to the probabilities of cross occupation. (B,D) Weak mutual interaction strength (*ν* = 2). Three stable states coexist (see inset). The same initial expression conditions—(*x*_0_, *y*_0_)—evolve towards an asymmetric (low,high) state.

How does autoregulation-based amplification work? We consider a mutual-activation circuit in an initial (transient) asymmetric expression state, i.e., *x*_0_≠*y*_0_, with *x*_0_, *y*_0_, being the concentration of each circuit component in non-dimensional units. We plot the time evolution of the species concentration (Figure 7A) and the probabilities of occupation of the associated binding sites—by the corresponding autoregulatory and crossregulatory species (Figure 7C). Despite the initial asymmetry, both components reach the same equilibrium expression (or present a monomodal distribution around this value, inset Figure 7A, when considering noisy gene expression [32]). The activation of the species with higher initial concentration leads to the increase in expression of the second component, mediated by the crossinteractions. This effect ultimately balances the probability of occupation of each binding site by their own species, which filters out the initial concentration disequilibrium (Figure 7C). Could the initial asymmetry be amplified, so that an asymmetric steady state is favored? When crossactivation is weaker (Figure 7B–D, *ν* = 2), the autointeraction of the species with smaller initial concentration does not become active. Autoregulation is only effective then on the species with higher concentration (Figure 7D). The circuit amplifies in this way the initial differences allowing the coexistence of symmetric and asymmetric equilibria (we now find three peaks in the distributions obtained by considering noisy gene expression, inset Figure 7B). Moreover, autoregulated-based compensation, by comparison, avoids expected unbalances in mutual-inhibition switches working in regimes with hardly active crossinteractions (small *σ*'s), since the autoregulation on both species dominates (Figure S8).

Multistability is however generally not expected in circuits exhibiting relatively strong mutual activation, as this might imply unrealistically strong autoregulation (Figure 2 and Figure S7). What would be then the influence of autoactivation? We suggest that it provides switches with a more flexible behavior. For instance, modulation of its strength can enlarge the bistable region, making the module respond to some external signals that would not be sensed otherwise (Figure S9). Autoregulation may change also the combinatory of signals to which a mutual-activation switch responds (Figure S10).

### Biological Scenarios of Progression and Decision Switches

How biologically relevant is the general framework that we have proposed? We investigated how these topologies enable different epigenetic regimes (the phenotypic map), characterized the molecular switches driving transitions between them, and discussed several information-processing features exhibited by these switches. To put these ideas in a biological context, we now first identify the presence of these regulatory architectures in specific scenarios, and then discuss several signal discrimination features that have been analyzed in these and related settings.

Indeed, we found a number of differentiation programs controlled by circuits constituted by two molecular regulators in a mutual-activation or mutual-inhibition configuration (Table 1). The main components of these circuits are usually master regulators, i.e., transcription factors involved in the determination of cellular fate, which exhibited autoactivation (a prevalent characteristic of master regulators, see Table S1). Interactions between module constituents are sometimes direct, normally transcriptional, or indirect, mediated by other molecular species. Recently studied examples include the embryonic stem-cell master regulators Oct4, Sox2, and Nanog [36]–[38]. These factors establish mutual-activation architectures between them—to maintain pluripotency—or mutual inhibition circuits, in combination with additional elements, to induce specific developmental fates [29],[39],[40]. Common instances of the latter involve Cdx2, promoting differentiation to trophectoderm [37], or Gata4/Gata6 linked to endodermal differentiation [39], see also [40].

Similarly, various stages of hematopoietic lineage specification are driven by modules exhibiting these architectures. In this situation, the presence of a third expression state, or priming state, is currently under inspection [12],[21],[22],[41]. In particular, genes in various lymphoid lineages are coexpressed at low levels in common lymphoid progenitors (CLPs) and in common myeloid progenitors (CMPs) [42],[43]. Specialization to different cell types from these common lineages proceeds through sequential steps where some genes are silenced and other activated [44],[45]. This is the case in B and T cell development (from CLP) [19],[46],[47] as well as in the macrophage/neutrophil decision (from CMP)—where a graded decision switch was proposed [11] with a similar architecture to the ones discussed here, but see additionally [12]. As in the previous case of embryonic stem cell differentiation, genes common to several lineages, i.e., at the top of the regulatory hierarchy, control the expression of other transcription factors involved in more specific lineage commitment. In summary, the collected scenarios in Table 1 suggest that the presence of mutual-activation circuits correlate with differentiation as a progression, while mutual-inhibition topologies appear mostly when alternative decisions from a precursor (priming) cellular state are made (Figure 1).

What sort of signal discrimination is found in these contexts? The influence of signal attributes in various developmental scenarios hints at the possibility that more elaborated signal processing could be at work. One example of this influence is the role of signal strength in thymocite differentiation [23],[48]. In particular, CD4/CD8 T-cell fate commitment is determined by the strength of the T-cell receptor signal, with strong and weak signals favoring either the CD4 and CD8 lineages, respectively [48]. Another interesting case of processing of stimulus strength is morphogen gradient interpretation [49]–[51]. In *Xenopus* mesoderm formation, activin, a member of the TGF-*β* family, acts on downstream genes in a concentration-dependent manner, with high concentrations inducing expression of the transcription factor Gsc and low concentrations activating the Xbra transcription factor, both regulating each other in a double negative feedback loop [51]. Similarly, a gradient of Shh signaling can be read by complementary complementary pairs of homeodomain proteins that cross-inhibit each other in a cell autonomous manner [52], specifying neural differentiation in the spinal cord. Finally, processing of signal duration has also been shown to be important in both T-cell fate commitment [53] and morphogen signalling [50]. The latter case is a good example of a more elaborated processing task in which Shh interpretation integrates both strength and duration of a signal to control differential gene expression.

Moreover, the precise temporal expression programs exhibited by genes involved in cell differentiation suggests that discrimination between signals at different times could be also important in these situations. For instance, during pancreas development (Table 1) one of the key factors promoting endocrine development (Foxa2) is already expressed in the first stage of development (the gut endoderm) but other essential factor (Sox9) appears later in the first pancreatic progenitor cells [54].

Signal processing ultimately leads to a fate decision. Two models, not necessarily exclusive, have been proposed. In a first model (sometimes termed ‘instructive’ or ‘selective’ regulation [25]), an external signal imposes the specific fate by activating or repressing a particular set of genes. This probably corresponds to the more standard scenario of how signaling determine fates. An alternative model is that in which a given fate is stochastically chosen among different pre-existing programs. An open issue in this latter model, and one that partially answers our analysis, is how the proportion between alternative stochastic fates is regulated in a population.

### Conclusions

What specific molecule is critical for this particular physiological response? This question is usually asked when a given cellular behavior comes under scrutiny. The search for such master regulators is specially relevant in the context of differentiation, where they become lineage specification factors, whose expression, or the lack of it, is associated to distinct cell fates. This approach, however, does not seem to be sufficient anymore. Indeed, an increasing number of studies confirm the view that regulators do not work in isolation, and that we need to study them as parts of genetic control circuits to properly recognize their function [9],[11],[17],[19],[37].

Even though the molecular components of such control circuits are obviously diverse, their architectures do exhibit two main unifying attributes. First, they represent a relatively simple positive loop structure, and second, this structure is constituted by interactions with a degree of sigmoidality (threshold-like action) that enables circuits to exhibit bistability [6]–[8]. Does the coexistence of more than two expression states lead to a fundamentally different type of regulation and signal processing? If so, how can we determine multistability and to what extent is this feature linked to more complex loop architectures?

To analyze these issues, we characterized the function of two-component circuits with the use of mathematical models. An additional property in these systems is that their main constituents are autoregulated. We made use of the phenotypic map, a parameter space characterizing the patterns of expression associated to these modules. Transitions between expression states were then considered to be induced by two major switch classes. A progression switch corresponds to a transition in which at most two expressions states should be available. Alternatively, a decision switch needs of three expression states, one before and two after the decision. Both types correspond to distinct bifurcations of the system equilibria [30]. This analysis also highlighted the fundamental role that autoregulation plays in these designs. Specifically, autoregulation in the mutual-inhibition circuit favors multistability, and thus decision switches, while it provides mutual-activation switches with more flexibility and enlarged stimulus reaction (but only two coexisting expression states, i.e., progression switch).

We examined a number of scenarios where master regulators and their interactions have been experimentally uncovered. We identified several architectures corresponding to the analyzed circuits, i.e., constituted by two principal autoregulated molecular agents in a mutual-activation/mutual-inhibition topology. Our study also provided an elaborated rationale of why master regulators largely exhibit autoregulation. In addition, we correlated mutual-activation/mutual-inhibition switches with differentiation as a progression or decision, respectively. These theoretical arguments helped thus to unify a wide range of biological data, and present progression and decision switches as fundamental design principles in the control of epigenetic differentiation.

We specially studied the abilities of these modules to respond and monitor stimuli, with special emphasis on decision switches. This revealed a series of findings. First, decision switches are able to elicit richer responses to differential signal parameters, like strength or duration, enhancing signal specificity [14],[24],[55]. Second, decision switches provide a circuit-based explanation to stochastic, but biased, cell fate determination [25],[26]. Identical cells can exhibit heterogeneous responses when experiencing similar external cues. These switches originate stochastic differentiation when an external stimulus is able to unstabilize the current expression state of an homogeneous population. The remaining system equilibria are then potentially reachable to each member of the population in a stochastic manner, e.g., due to biochemical noise [32]. The population distribution of gene expression can be further modulated by any asymmetry presented in the signal or the circuit main characteristics. Last, decision switches appear as a module able to process delayed signals. In particular, we showed how this switch can implement two-interval discrimination tasks. This capability allows cells to adapt to varying environments by holding the history of a previous condition in a kind of short-term memory (working memory). Cells would modify their identities by comparing the first conditions with those found in a second environment. Some of these discrimination performances are similar to the one found in cortical circuits in monkeys, where neural network models of mutual inhibition with recurrent self-excitation have been hypothesized to mediate these decisions [27],[28], which emphasizes the presence of similar dynamical principles in circuits underlying apparently non-related biological functions [34]. The presence of a state of working memory also offers an alternative mechanism to the dynamics of transient gene expression. While most studies focused on the role of DNA structural modifications to transform these short-term states into stable long-term memories, [56],[57] for two recent examples, decision switches would accomplish a similar function by means of feedback regulation.

## Materials and Methods

To derive the mathematical models used in this study, we consider all biochemical reactions involved in transcription regulation and expression of two interacting genes (dimerization reactions, binding/unbinding of transcription factors to promoters, transcription, translation and degradation, see Text S1 for details). Separation of time scales and standard quasi-steady state assumptions lead to the following model for the time evolution of the two gene products:
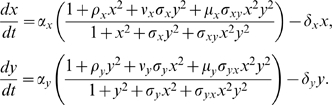
(1)Here, *x*, *y* describe protein concentrations in non-dimensional form. We introduce two types of parameter sets. One is linked to the activation/inhibition strengths in units of basalproduction, i.e., *ρ_i_*, *ν_i_*, *μ_i_* with *i* = {*x*, *y*}. Specific ranges of *ν* determine the various modules under study, i.e, mutual inhibition with *ν*∈[0,1), or mutual activation with *ν*>1. The second parameter group is associated to the protein threshold values required for an interaction, i.e., its response, to become active. Specifically, the *σ*'s quantify the ratio of response threshold of each link, e.g., ratio of binding affinities in the case of transcriptional interactions. In addition, *α_i_* and *δ_i_* correspond to the basal production and degradation rate, respectively. We further assume a symmetric parametric regime (parameters equal in both species, e.g., *ρ* = *ρ_x_* = *ρ_y_*), and that *σ_xy_* = 0, i.e., non-cooperative interactions (to see the role of cooperativity see Figure S3C and S3D). Finally, we scale the time by the corresponding protein degradation rate which results in an average production rate, *a* = *α*/*δ* (note also that larger Hill coefficient implies a higher degree of non-linearity but qualitative conclusions hold, Figure S11). These parameters can be quantified experimentally, and represent a minimal set of biologically relevant features able to characterize circuit behavior.

Phenotypic maps are obtained numerically by sampling the parameter space with different initial conditions and letting the system to reach all available steady states. Moreover, stochastic gene expression is simulated by using the Gillespie's algorithm in most cases (taking into account mRNA dynamics, see Equation 7 in Text S1). For the specific situation of signal discrimination, a Langevin method was implemented to reduce computational time (Text S1 for details).

## Supporting Information

Figure S1 Deterministic dynamics for the reduced two-variable model (black lines) compared to the four-variable model with different ratios of mRNA and protein degradation (Δ). Red line: Δ = 10. Blue line: Δ = 1. Solid lines correspond to the time evolution of the x component, and dashed lines to the y component. The system is in the tri-stability regime with ρ_x_ = ρ_y_ = 10, ν_x_ = ν_y_ = 0 (mutual inhibition), σ_x_ = σ_y_ = 0.2 and α_x_ = α_y_ = 1.
(0.88 MB EPS)

Figure S2 Comparison of different algorithms and intrinsic noise sources. Probability distribution of the x component concentration for a population of cells in a symmetric high expression state. Solid lines: simulations with Gillespie's algorithm. Dashed lines: solution of the chemical Langevin equations. Black lines correspond to a burst parameter b = 1 and a volume factor Ω = 10 (V = V_0_·Ω). Blue lines: effect of translational bursting (b = 10, Ω = 10). Red lines: Effect of finite size noise (b = 1, Ω = 1). Other parameters of the model for the stochastic simulations are ρ_x_ = ρ_y_ = 10, ν_x_ = ν_y_ = 0, σ_x_ = σ_y_ = 0.2 (mutual inhibition, tri-stability regime), α_x_ = α_y_ = 1 and k^x^_x_ = k^y^_y_ = 0.001 nM^−2^.
(0.79 MB EPS)

Figure S3 (A,B). Phenotypic map of the circuit with average production rate a = 1 and different cross-interaction strengths. (A) ν = 2, (B) ν = 20 (the cross-inhibition case ν = 0 is shown in Fig. 2B). In these panels, like in Figure 2, promoters are completely overlapping (σ_xy_ = 0). (C,D) A possible role played by cooperativity among species. Here we plot the phenotypic map for a = 1, as a function of the autoregulation and the joint interaction strength parameter μ, Eq. (1) main text, for slightly non-overlapping promoters (σ_xy_ = 0.001) and cross-interaction strengths ν = 2 (C) and ν = 20 (D). In the case of total competition for the same promoter site, panels (A,B), positive cross-interaction is not able to generate bistability of symmetric expression states (0,0), (1,1), since at an average production rate a = 1 the lower (0,0) state is not stabilized. Strong cooperativity (recall that μ = ρ×ν for independent regulation) together with competition for the same binding sites favors the appearance of a low (0,0) expression state and bistability (stability regions correspond to the areas inside cusps).
(1.34 MB EPS)

Figure S4 Reversible (graded) deci-switch. The intersection between the circuit response curves or nullclines (lines in the x-y planes) identifies the system steady states, being these either stable (filled circles), or unstable (empty circles). In this way, a range of different initial concentrations of the circuit components (basin of attraction; light and dark grey areas) ends up in the same expression state. A reversible deci-switch is associated to a transition in which the initial expression state (0,0) becomes unstable (A). Two new asymmetric states appear in a graded fashion (B). This is a supercritical pitchfork bifurcation, insets (A–B), where the magnitude and types of available equilibria are plotted as a given parameter changes in the x-axis (solid line; steady state, dotted line; unstable state). Note that in this case there exist no hysteresis. The transition is reversible, which means that the appearance of new expression states strongly depends on the presence of a external factor (acting as bifurcation parameter). This could represent, for instance, a primary master regulator.
(0.78 MB EPS)

Figure S5 (A) Increased autoregulation enhances duration detection. Here we examine how the response of a decision switch to stimulus duration depends on autoregulation strength. The response for an autoregulation strength ρ = 10 (red line and filled circles, the same as in Fig. 4B) is compared to the response at ρ = 50 (blue line, open squares) for a fast signal producing the same threshold in duration detection. Larger autoregulation induces a sharper discrimination performance. Other parameter values are ν = 0, a = 1. (B) Increased autoregulation, however, delays differential amplitude detection in stochastic decision switches. Same symbols and parameters than those in panel (A).
(1.10 MB EPS)

Figure S6 Effect of fast and slow signals on strength discrimination. A mutual inhibition switch is placed in a regime (ρ = 30, ν = 0, σ = 0.2, a = 0.1) where a symmetric (high,high) expression state becomes unstable with similar amplitudes for: A. fast and B. slow degradation signals. Red lines and circles show the performance using deterministic signal pulses, and blue lines (squares) adding noise to the signals such that the mean number of signal molecules is the same in both cases. Lines are fits to Weibull or stretched exponential functions.
(1.15 MB EPS)

Figure S7 Multistability domains as a function of relative interaction strength (a = 1). For moderate to large average production rates and autoregulation strengths, the boundaries between monostable and multistable domains follow a linear relation, ρ/ν∝1/σ. For instance, ρ/ν>20 indicates a tri-stable domain at σ = 0.2. Notice that for high σ values the symmetric expression state (1,1) is no longer available and only two asymmetric equilibria coexist.
(0.84 MB EPS)

Figure S8 Autoregulation as a compensation mechanism. For mutual inhibition (ν = 0) and moderate autoactivation (ρ = 5), the ratio of binding affinities (σ parameter) determines if the circuit behaves as a toggle switch (A,C) or generates tri-stability (B,D). (A) With similar binding affinities (σ = 0.6), the autoregulation is acting at the same time than cross-interaction. Then mutual inhibition dominates, amplifying the expression of the ‘winner’ species in detriment of the ‘looser’ one. In this regime, only two asymmetric states exist [(low,high), (high,low)]. This is illustrated in the inset by the probability distribution of the x component, obtained by solving the stochastic system. (C) The probability of promoter occupation for autoactivation of the looser species (in this case, x-auto, black solid line) never reaches the necessary level for effective activation. (B) If relative binding affinity is strongly favored for autoactivation (σ = 0.2), the species with smaller initial expression is rapidly increased, compensating the initial difference. Here a new (high,high) expression state is available compared to the previous case (see inset). (D) Probability of occupation for autoregulation is increased faster in the less abundant species (black solid line).
(1.14 MB EPS)

Figure S9 Role of positive autoregulation in mutual-activation switches. (A) Response to a signal increasing the degradation of both components as a function of cross-interaction strength, for a switch without autoregulation (a = 1). The system is initially (no stimulus) in a monostable high expression regime. For ν<12, the signal decreases gradually the expression. For higher ν values, a low expression state is also stabilized and a progression (1,1)→(0,0) can take place depending on signal strength. (B) For the same stimulus, we take a cross-interaction strength of ν = 10 (no response regime) and examine the response as a function of autoregulation. For ρ>10 a bistability regime, and eventually a progre-switch, transition can take place. Thus, the presence of autoregulation enables a circuit to work as a switch in a signaling environment where it would not work as such otherwise, i.t., without autoactivation. Other parameters in (B) are a = 1, σ = 0.2.
(1.65 MB EPS)

Figure S10 Autoregulation favors flexibility in signal processing. Normalized response after a sustained degradation signal with different intensities in x and y components. (A) A switch without autoregulation in a symmetric bistable regime (a = 0.1, ν = 20) responds to signal asymmetries. In a color code, we show the concentration of the x species normalized by the initial equilibrium value (no signal). Note that for signal larger than {similar, tilde operator } 0.1 in one component a transition (1,1)→(0,0) is attained, irrespective of the strength of the signal in the other component. (B) For the same value of crossinteraction (ν = 20) but strong autoregulation (ρ = 20, σ = 0.1, see Figure 2 in main text) a switch transition requires higher signal strengths and a minimum signal threshold in both components.
(1.50 MB EPS)

Figure S11 Influence of Hill coefficients on phenotypic maps. This map shows the areas of coexistence of several expression states (multistability) in a σ-ρ parameter space for a Hill coefficient of n = 4, e.g., x, y species acting as tetramers. These regions are: I_L_; one (low,low) expression state, II_A_; coexistence of (low,high)-(high,low), antisymmetric, expression states, III_{L,H}_; tri-stability with two antisymmetric states and one symmetric state, low or high, IV; coexistence of four expression states. (A) Phenotypic map for mutual-inhibition with low basal production rate (ν = 0, a = 0.1) corresponding to Fig. 2A in main text. (B) Phenotypic map for mutual inhibition and higher basal production (ν = 0, a = 1) corresponding to Fig. 2D, main text. Note that larger Hill coefficient implies a higher degree of non-linearity but that qualitative conclusions hold, e.g., there exits a decision switch transition from IV→III_L_ if the initial expression state is (high,high), Fig. S11.A.
(1.21 MB EPS)

Table S1 Positive autoregulation of the factors involved in mutual activation/inhibition architectures in Table 1.
(0.05 MB PDF)

Text S1 Further discussions on the mathematical models and the approximations considered.
(0.12 MB PDF)
