# Supplementary material for: Multistable Decision Switches for Flexible Control of Epigenetic Differentiation
Source: PLoS Comput Biol. 2008 Nov 28;4(11):e1000235. doi: 10.1371/journal.pcbi.1000235 (PMC2580821; doi:10.1371/journal.pcbi.1000235)
Supplement: Table S1 — Positive autoregulation of the factors involved in mutual activation/inhibition architectures in Table 1. (0.05 MB PDF) [file pcbi.1000235.s012.pdf]

Factor	*Type of autoregulation*	*Mediators*	*Reference*
Oct4/Sox2	Transcriptional cooperative	Sox2/Oct4	Chew [1]
Nanog	Transcriptional cooperative	Sall4	Boyer [2]<br>Wu [3]
Ac	Transcriptional cooperative	Da	Cabrera [4]<br>Van Doren [5]
Sc	Transcriptional cooperative	Da	Martinez [6]<br>Van Doren [5]
MyoG	Transcriptional	Myf	Blais [7]<br>Braun [8]
Mef2C	Transcriptional	MyoD	Blais [7]<br>Wang [9]
Sox9	Transcriptional	Unknown	Lynn [10]
Foxa2	Transcriptional	Direct	Pani [11]
p42	Post-translational	Mos, MEK	Matten [12]
Cdc2	Post-translational	Cdc25, Myt1	Xiong [13]
Casp3	Post-translational	Casp8	Legewie [14]
Casp9	Post-translational	Apf1	Shiozaki [15]
Cdx2	Transcriptional	Cell type specific	Xu [16]

Factor	*Type of autoregulation*	*Mediators*	*Reference*
GATA1	Transcriptional	Direct	Tsai [17]
PU.1	Transcriptional cooperative	cJun	Okuno [18]
T-bet	Transcriptional + Autocrine loop	cytokines(IFN-γ)	Mullen [19] Lighvani [20]
Gata3	Transcriptional+ Autocrine loop	IL-4	Zhou [21] Ansel [22]
Pax6	Transcriptional	Direct	Yamaguchi [23]
Pax2	Transcriptional	Shh signal?	Schwarz [24]
cog-1	Transcriptional	Direct	Johnston [25]
die-1	Translational	lim-6	Johnston [25]
wts	Transcriptional	Unknown	Mikeladzi-Dvali [26]
melt	Transcriptional	Unknown	Mikeladzi-Dvali [26]

References

[1] Chew J.-L. et al. (2005) *Mol. Cell. Biol.* **25** 6031-6046

[2] Boyer L. A. *et al.* (2005) *Cell* **122** 947-56.

[3] Wu Q. et al. (2006) *J. Biol. Chem.* **281** 24090-24094.

[4] Cabrera C. V. and Alonso M. C. (1991) *EMBO J.* **10** 2965-2973.

[5] Van Doren M., Powell P. A., Pasternak D., Singson A. and Posakony J. W. (1992) *Genes Dev.* **6** 2592-2605.

[6] Martinez C. and Modolell J. (1991) *Science* **251** 1485-1487.

[7] Blais, A., Tsikitis, M., Acosta-Alvear, D., Sharan, R., Kluger, Y., and Dynlacht. B. D. (2005) *Genes & Dev.* **19** 553-569.

[8] Braun T., Bober E., Buschhausen-Denker G., Kohtz S., Grzeschik K. H., Arnold H. H. and Kotz S. (1989) *EMBO J.* **8** 36173625.

[9] Wang D. Z., Valdez M. R., McAnally J., Richardson J. and Olson E. N. (2001) *Development* **128** 4623-4633.

[10] Lynn, F. C., Smith, S. B., Wilson, M. E., Yang, K. Y., Nekrep, N., and German, M. S. (2007) *Proc. Natl. Acad. Sci. USA* **104** 10500-05.

[11] Pani L., Qian X. B., Clevidence D. and Costa R. H. (1992) *Mol. Cell. Biol.* **12** 552-62.

[12] Matten W. T., Copeland T. D., Ahn N. G. and Vande Woude G. F. (1996) *Devel. Biol.* **179** 485-92

[13] Xiong, W., and Ferrell, J. E. Jr. (2003) *Nature* **426** 460-465.

[14] Legewie S., Blüthgen N. and Herzel H. (2006) *PLoS Comp. Biol.* **2** 1061-1073.

[15] Shiozaki E. N., Chai J. and Shi Y. (2002) *Proc. Natl. Acad. Sci. USA* **99** 4197-4202.

[16] Xu, F., Li, H., and Jin, T. (1999) *J. Biol. Chem.* **274** 34310-34316.

[17] Tsai S. F., Strauss E. and Orkin S. H. (1991) *Genes Dev.* **5** 919-931.

[18] Okuno Y. et al (2005) *Mol. Cell. Biol.* **25** 2832-2845.

[19] Mullen A. C. *et al.* (2001) *Science* **292** 1907-1910.

[20] Lighvani A. A. *et al.* (2001) *Proc. Natl. Acad. Sci. USA* **98** 15137-15142.

[21] Zhou, M., and Ouyang, W. (2003) *Immunol. Res.* **28**, 25-37.

[22] Ansel K. M., Lee D. U. and Rao A. (2003) *Nat. Immun.* **4** 616-623.

[23] Yamaguchi, Y., Sawada, J., Yamada, M., Handa, H., and Azuma, N. (1997) *Genes Cells* **2**, 255-261.

[24] Schwratz M. et al. (2000) *Development* **127** 4325-4334.

[25] Johnston, R. J. Jr., Chang, S., Etchberger, J. F., Ortiz, C. O., and Hobert O. (2005) *Proc. Natl. Acad. Sci. USA* **102** 12449-12454.

[26] Mikeladze-Dvali T., Wernet, M. F., Pistillo, D., Mazzoni E. O., Teleman, A. A., Chen Y., Cohen, S., and Desplan C. (2005) *Cell* **122** 775-787.
