# Supplementary material for: Multistable Decision Switches for Flexible Control of Epigenetic Differentiation
Source: PLoS Comput Biol. 2008 Nov 28;4(11):e1000235. doi: 10.1371/journal.pcbi.1000235 (PMC2580821; doi:10.1371/journal.pcbi.1000235)
Supplement: Text S1 — Further discussions on the mathematical models and the approximations considered. (0.12 MB PDF) [file pcbi.1000235.s013.pdf]

Supplement

Multistable decision switches for flexible control of epigenetic differentiation.

Raúl Guantes[1,2] and Juan F. Poyatos[3]

[1] Department of Condensed Matter Physics and
[2] Instituto Nicolás Cabrera, Facultad de Ciencias C-XVI, Universidad Autónoma de Madrid, Madrid, Spain.
[3] Logic of Genomic Systems Laboratory, Spanish National Biotechnology Centre, Consejo Superior de Investigaciones Científicas (CSIC), 28049 Madrid, Spain.

Effective models; motivation

To describe the dynamics of many of the control circuits listed in Table 1 (main text), we consider two interacting regulators which are able to affect each other's production, while positively autoregulating their own expression. We further suppose these regulators to be transcription factors acting as homodimers, as is the case in bHLH proteins (e.g. Ac, Sc, Pax6, MyoG in Tables 1 and S1), leucine zipper factors or some types of homeodomain proteins, such as the POU factor Oct4 [1] or the caudal related protein Cdx2 [2]. Although many of the feedback interactions found in cell differentiation contexts are mediated by additional transcription factors, signals, etc., we seek to represent the basic dynamical features of these switches with a plausible mathematical model and a minimal set of parameters of biological significance. For instance, indirect regulation with the aid of an intermediate species can be simplified, under the assumption that this intermediate is in equilibrium, to the same mathematical equations discussed here. Thus, we start by writing the biochemical reactions for production and degradation of four molecular species: protein and mRNA of each of the two components (X, Y) of the control module. These reactions can be separated in fast and slow.

Fast equilibrium reactions

We assume that DNA-binding reactions and dimerization are fast. Furthermore, they are lumped together in a single reaction term under the equilibrium assumption. Since each component is regulated by its own product as well as by the

partner's, we consider in principle different promoter binding sites for X or Y molecules. These sites can be independent, interacting or completely overlapping as is the case in both prokaryotic [3] and eukaryotic transcription regulation [4], giving rise to a potential combinatorial logic of gene expression [5,6].

$$
\begin{aligned}
P_x + 2X &\overset{K_x^x}{\longleftrightarrow} P_xX_2, \\
P_x + 2Y &\overset{K_y^x}{\longleftrightarrow} P_xY_2, \\
P_y + 2Y &\overset{K_y^y}{\longleftrightarrow} P_yY_2, \\
P_y + 2X &\overset{K_x^y}{\longleftrightarrow} P_yX_2,
\end{aligned}
\tag{1}
$$

$$
\begin{aligned}
P_x + 2X + 2Y &\overset{K_{xy}^x}{\longleftrightarrow} P_xX_2Y_2, \\
P_y + 2X + 2Y &\overset{K_{xy}^y}{\longleftrightarrow} P_yX_2Y_2.
\end{aligned}
\tag{2}
$$

Here the K's represent products of equilibrium constants, being this the product of binding (protein-DNA) and dimerization constants, in Eqs. (1), or joint binding constants, $K_{xy}^i (i = x, y)$ in Eqs. (2). In the case of two independent promoter sites, $K_{xy}^i = K_x^i \times K_y^i$, while for overlapping promoters, only one species can be bound at a time and $K_{xy}^i = 0$.

Slow equations

Transcription, translation and degradation of both components are considered as slow reactions. Explicitly,

$$
\begin{aligned}
P_x &\overset{\beta_x}{\longrightarrow} P_x + m_x, \\
P_xX_2 &\overset{\rho_x\beta_x}{\longrightarrow} P_xX_2 + m_x, \\
P_xY_2 &\overset{\nu_x\beta_x}{\longrightarrow} P_xY_2 + m_x, \\
P_xX_2Y_2 &\overset{\mu_x\beta_x}{\longrightarrow} P_xX_2Y_2 + m_x, \\
m_x &\overset{\gamma_x}{\longrightarrow} m_x + X, \\
m_x &\overset{\delta_{mx}}{\longrightarrow} \emptyset, \\
X &\overset{\delta_x}{\longrightarrow} \emptyset,
\end{aligned}
\tag{3}
$$

and analogous equations for the Y species. Here $\beta_i (i = x, y)$ are the corresponding basal transcription rates, ρ_i and ν_i the auto and crossregulation strengths respectively, and μ_i the joint regulatory strength. For independent promoter sites $\mu_i = \rho_i \times \nu_i$. With the constraint that the total number of promoters (copy number) is fixed, $P_i^T = P_i + P_i X + P_i Y_2 + P_i X_2 Y_2$, and using the fast equilibrium reactions [Eqs. (1)-(2)] we obtain the time evolution of the four molecular species considered here as

$$\begin{aligned}
\frac{dm_x}{dt} &= \beta_x P_x^T \frac{1 + \rho_x K_x^x \cdot X^2 + \nu_x K_y^x \cdot Y^2 + \mu_x K_{xy}^x \cdot X^2 \cdot Y^2}{1 + K_x^x \cdot X^2 + K_y^x \cdot Y^2 + K_{xy}^x \cdot X^2 \cdot Y^2} - \delta_{mx} m_x, \\
\frac{dX}{dt} &= \gamma_x m_x - \delta_x X, \\
\frac{dm_y}{dt} &= \beta_y P_y^T \frac{1 + \rho_y K_y^y \cdot Y^2 + \nu_y K_x^y \cdot X^2 + \mu_x K_{xy}^x \cdot X^2 \cdot Y^2}{1 + K_x^y \cdot X^2 + K_y^y \cdot Y^2 + K_{xy}^y \cdot X^2 \cdot Y^2} - \delta_{my} m_y, \\
\frac{dY}{dt} &= \gamma_y m_y - \delta_y Y.
\end{aligned} \tag{4}$$

These are differential equations for the total number of proteins per cell. To convert to cellular protein concentration one needs to divide by cell volume, $[X] = X/V$. The time evolution for each species concentration is now

$$\begin{aligned}
\frac{d[m_x]}{dt} &= \beta_x [P_x^T] \frac{1 + \rho_x k_x^x \cdot [X^2] + \nu_x k_y^x \cdot [Y^2] + \mu_x k_{xy}^x \cdot [X^2] \cdot [Y^2]}{1 + k_x^x \cdot [X^2] + k_y^x \cdot [Y^2] + k_{xy}^x \cdot [X^2] \cdot [Y^2]} - \delta_{mx} [m_x], \\
\frac{d[X]}{dt} &= \gamma_x [m_x] - \delta_x [X], \\
\frac{d[m_y]}{dt} &= \beta_y [P_y^T] \frac{1 + \rho_y k_y^y \cdot [Y^2] + \nu_y k_x^y \cdot [X^2] + \mu_x k_{xy}^x \cdot [X^2] \cdot [Y^2]}{1 + k_x^y \cdot [X^2] + k_y^y \cdot [Y^2] + k_{xy}^y \cdot [X^2] \cdot [Y^2]} - \delta_{my} [m_y], \\
\frac{d[Y]}{dt} &= \gamma_y [m_y] - \delta_y [Y],
\end{aligned} \tag{5}$$

where $[P_i^T]$ is the total promoter concentration for each gene, and the deterministic rate constants have been appropriately rescaled by cell volume for the series of bimolecular reactions, i.e., $k_j^i = K_j^i \times V^2$ and $k_{ij}^i = K_{ij}^i \times V^4$, with $\{i, j\} = \{x, y\}$. Cell growth and division can be taken into account in an approximate manner by assuming an exponential law for growth [8], $V(t) = V_0 \exp(k_g t)$. The growth rate is given by $k_g = \ln(\alpha)/T_{\text{ccyc}}$, where T_{ccyc} is the mean division time and α a

scale parameter giving the maximum increase in cell volume (typically $\alpha = 2$). Then cell division occurs when $V(t = T_{\rm ccyc}) = \alpha V_0$. Assuming that the total number of promoters scales proportionally with the cell volume, and using for the time evolution of the molecular concentrations $\frac{d[X]}{dt} = \frac{1}{V(t)}\left(\frac{dX}{dt} - [X]\frac{dV(t)}{dt}\right)$, the resultant equations are identical to Eqs. (5) except that the degradation rates for mRNA and protein are now given by $\delta'_{mx,my} = \delta_{mx,my} + k_g$, $\delta'_{x,y} = \delta_{x,y} + k_g$, which are effectively taken as a single parameter. For stable proteins, $\delta_{x,y} \ll k_g$ and degradation is just due to dilution by cell growth and division. Protein degradation can be faster (e.g., by proteases or by sequestration or inactivation with different molecular species), which implies that $\delta_{x,y} \gg k_g$ and the growth term can be neglected.

Scaled variables and quasi-steady state approximation

We want to reduce both the number of variables to make the system amenable for mathematical and phase plane analysis, and the number of parameters to the minimum set with biological significance. First, it is convenient to define dimensionless mRNA and protein concentrations by

$$\begin{aligned} x &= \sqrt{k_x^x}[X], \\ m_x &= \sqrt{k_x^x}[m_x], \\ y &= \sqrt{k_y^y}[Y], \\ m_y &= \sqrt{k_y^y}[m_y]. \end{aligned} \tag{6}$$

Eqs. (5) now become

$$\begin{aligned} \frac{dm_x}{dt} &= \beta_x\sqrt{k_x^x}[P_x^T]\frac{1 + \rho_x \cdot x^2 + \nu_x\sigma_x \cdot y^2 + \mu_x\sigma_{xy} \cdot x^2 \cdot y^2}{1 + x^2 + \sigma_x \cdot y^2 + \sigma_{xy} \cdot x^2 \cdot y^2} - \delta_{mx}m_x, \\ \frac{dx}{dt} &= \gamma_x m_x - \delta_x x, \\ \frac{dm_y}{dt} &= \beta_y\sqrt{k_y^y}[P_y^T]\frac{1 + \rho_y \cdot y^2 + \nu_y\sigma_y \cdot x^2 + \mu_y\sigma_{yx} \cdot x^2 \cdot y^2}{1 + y^2 + \sigma_y \cdot x^2 + \sigma_{yx} \cdot x^2 \cdot y^2} - \delta_{my}m_y, \\ \frac{dy}{dt} &= \gamma_y m_y - \delta_y y, \end{aligned} \tag{7}$$

with $\sigma_i = k_j^i/k_i^i$ (ratio of cross over self-binding rates) and $\sigma_{ij} = k_{ij}^i/(k_i^i \cdot k_j^i)$ (ratio of joint over independent rates for simultaneous binding of both species).

We note that for completely overlapping promoter sites (`XOR` transcriptional gate) $\sigma_{ij} = 0$, whereas for independent promoter sites $\sigma_{ij} = \sigma_i \times \sigma_j$, $\mu_i = \rho_i \times \nu_i$ and the non-linear production rates for mRNA are the product of two Hill functions, one for each variable. Applying the standard quasi-steady state approximation (QSSA, fast mRNA dynamics compared to protein dynamics), $\frac{dm_x}{dt} = \frac{dm_y}{dt} \sim 0$, one readily obtains Eqs. (1) in the main text with

$$\alpha_i = \beta_i [P_i^T] \sqrt{k_i^i}\, b_i, \tag{8}$$

where $b_i = \gamma_i / \delta_{mi}$ is the burst parameter or translational efficiency.

To gain insight on the validity of this approximation, let as assume similar production and degradation rates for both components, i.e., $\gamma_x = \gamma_y \equiv \gamma$, $\beta_x = \beta_y \equiv \beta$, $\delta_x = \delta_y \equiv \delta$ and $\delta_{mx} = \delta_{my} \equiv \delta_m$. Rescaling time by the protein degradation rate, $\tau = t \cdot \delta$, Eqs. (7) can be written as

$$\begin{aligned}
\frac{dm_x}{d\tau} &= \frac{\alpha_x}{b\,\delta} f(x,y) - \Delta \cdot m_x, \\
\frac{dx}{d\tau} &= \Delta \cdot b \cdot m_x - x, \\
\frac{dm_y}{d\tau} &= \frac{\alpha_y}{b\,\delta} g(x,y) - \Delta \cdot m_y, \\
\frac{dy}{d\tau} &= \Delta \cdot b \cdot m_y - y,
\end{aligned} \tag{9}$$

where $\Delta = \delta_m / \delta$ is the relative messenger-protein degradation rate. The variation of mRNA at short time scales can be obtained by considering the protein concentration fixed. Then, from Eqs. (9) one sees that for constant protein values mRNA decays as $m_{x,y}(\tau) \propto \exp(-\Delta\tau)$ while for constant messenger concentration the decay in protein concentration is simply $\propto \exp(-\tau)$. Therefore it is reasonable to expect that mRNA dynamics adapts much faster to protein changes when $\Delta >> 1$. This is illustrated in Fig. S1. For $\Delta = 10$, the QSSA is a fairly good approximation to the four-dimensional dynamics (compare black and red lines), while for $\Delta = 1$ the two- and four-dimensional trajectories (black and blue lines) separate, although they eventually converge to the same equilibrium state in the long run. Indeed, we checked that the multistability domains in the phenotypic maps shown in Figures 2 and S3 are the same when calculated with the four-variable model [11]. The effect of changing b is important when considering the stochastic dynamics [9]. In fact, experimental studies of stochastic gene expression in single cells demonstrate that translational bursting is an important

source of stochasticity in prokarytic [10] and eukaryotic gene expression when coupled to transcriptional noise [12].

Stochastic simulations and parameter ranges

Provided that equilibrium and quasi-steady state approximations hold for the deterministic system, stochastic simulations using Gillespie's method [13] can be simplified in a similar vein [14, 15]. Therefore we used the four-dimensional model Eqs. (5) as a starting point for the stochastic simulations. To have a full correspondence with the simplified two-dimensional model, Eq. (1) in main text, we need to specify the following parameters:

(1) Regulatory strengths (ρ_i, ν_i, μ_i), or fold-change in promoter activity once the proper transcription factor is bound. These parameters span from 0 (total inhibition) to 100 for the case of strong activation. This range reflects orders of magnitude typically found in both prokayotic and eukaryotic gene regulation.

(2) Relative binding affinities both for individual(σ_i) and joint (σ^i_{xy}) species. σ_i is varied between (0.01,2). A value of $\sigma_i < 1$ denotes higher binding affinity of a given promoter for its own protein (and then the threshold for autoregulation is smaller than for cross-interaction) while $\sigma_i > 1$ corresponds to the opposite situation. σ^i_{xy} is set to 0 for most of the paper calculations (completely overlapping promoter sites) but it can range from this value to $\sigma_i \times \sigma_j$ (independent promoter sites).

(3) We need also to specify the scaling factors k^x_x and k^y_y (promoter binding constants for each species) in the deterministic case. Recall that these are the product of dimerization and protein-DNA association constants. Here we fix $k^x_x = k^y_y = 10^{-3}$ nM^{-2} (a range $10^{-1} - 10^{-3}$ is typically found in bacteria [16]). The stochastic rate constants are obtained from the deterministic ones as $K^x_x = k^x_x/V^2$, $K^y_y = k^y_y/V^2$, $K^x_y = K^x_x \cdot \sigma_x$, $K^y_x = K^y_y \cdot \sigma_y$, $K^x_{xy} = K^x_x \cdot K^x_y \cdot \sigma_{xy}$ and $K^y_{xy} = K^y_y \cdot K^y_x \cdot \sigma_{yx}$, where V represents cell volume. In the stochastic simulations cell growth and division can be taken into account by considering V as an additional random variable obeying the equation $dV/dt = k_g V$, until a maximum value is attained [17,18]. Here we do not take into account the contribution of cell growth to gene expression noise, and fix $V = V_0 \cdot \Omega$ in stochastic simulations, where V_0 is a reference cell volume including Avogadro's number and Ω a scale factor giving the

contribution of protein number or finite size effects to molecular noise [19]. For a given value of molecular concentrations, the greater the scaling factor Ω the larger the number of molecules, i.e., noise is reduced. In our simulations, we take $V_0 = 10^9$l for simplicity (note that concentrations are in nM) which implies a cell volume of 1.7 μm^3. We also take $\Omega = 10$ in most of the simulations. The effect of finite size noise in the protein steady-state distributions is illustrated in Figure S2 (compare black and red lines).

(4) Relative molecular stabilities. From the adimensional analysis of the four-variable model, Eqs. (9), we also fix the ratio of protein/mRNA degradation to a value $\Delta = 10$, which implies a ten times faster degradation rate for mRNAs than for protein. Then we suppose that proteins are stable and mainly decay by dilution due to cell growth. Assuming a protein half-life of one hour ($\delta_x = \delta_y = 1$ h^{-1}) then messengers are degraded on average every six minutes, which are typical values of prokaryotes.

(5) Burst parameter or translational efficiency. We use $b_x = b_y = 1$ for most simulations (for a glimpse on the effect induced by translational efficiency on molecular noise, compare black and blue lines in Figure S2).

(6) Finally, we specify the average protein production rate $a_i = \alpha_i/\delta_i$ as for the two-dimensional model Eq. (1). Setting the copy-number $P_x^T = P_y^T = 1$, then the basal transcription rates follow from the given parameters as $\beta_i = a_i \cdot \delta_i/(\sqrt{K_i^i} \cdot P_i^T \cdot b_i)$. Note that a ten-fold increase in translational efficiency is coupled here to a ten-fold decrease in transcription and thus the average number of proteins remains the same, but the number of messenger molecules is reduced (mRNA noise). On the other hand, a ten-fold increase in volume produces also a ten-fold increase in transcription and abundance of proteins and mRNAs are increased in the same way (finite size noise). The different effects of these two factors in the width of protein distributions are shown in Figure S2.

Signals

We model signals as molecular species external to the circuits considered. As such, they follow an independent dynamics that we approximate as a birth-death process:

$$\frac{dS}{dt} = \alpha_s - \delta_s \cdot S. \tag{10}$$

To create a signal pulse, we fix the degradation rate at $\delta_s = 1h^{-1}$. Initially, the signal production rate is $\alpha_s = 0$. At a given time, the signal is produced at a steady rate $\alpha_s = s_{eq} \cdot \delta_s$ so that it quickly reaches their maximum value s_{eq} (see insets in Fig. 5A). The signal is terminated at a later time when the source disappears and α_s is set to zero again. For the deterministic equations, s is the signal concentration and α_S has units of $nM.h^{-1}$. In the stochastic simulations, the noise in the signal can be varied independently from the noise in the circuit components defining the stochastic production rate $\alpha_s^s = \alpha_s \cdot V_s$ where V_s is a volume factor changing the number of signal molecules. Signals are broadly considered affecting the circuit components in two possible ways: fast, inducing post-translational modifications of the X or Y proteins. In this case, the equations for (X, Y) are only modified by an extra linear degradation/production term originated from the reactions

$$\begin{aligned} S + X &\xrightarrow{\delta_x^s} S, \\ S + Y &\xrightarrow{\delta_y^s} S, \\ S &\xrightarrow{\alpha_x^s} S + X, \\ S &\xrightarrow{\alpha_x^s} S + Y. \end{aligned} \tag{11}$$

Slow: signals may be transcription factors external to the circuit, that may act repressing or activating the (X, Y) components. Here we assume that independent promoter sites are available for the signal molecules (although they could also operate cooperatively with any of both species). Equations are now modified according to the reactions.

$$\begin{aligned} P_x S &\xrightarrow{\tau_x \beta_y} P_x S + m_x, \\ P_y S &\xrightarrow{\tau_y \beta_y} P_y S + m_y, \end{aligned} \tag{12}$$

where $\tau_{x,y}$ are the signal regulation strengths ($\tau_{x,y} < 1$ for inhibitory signals, $\tau_{x,y} > 1$ for excitatory ones).

In the main text, we showed examples of signal processing with fast or post-translational events. Similar computational properties can be obtained with slow or transcriptional signals in the appropriate regimes. However, slow signals may change the response dynamics and thus the discrimination performance of some stimulus features. An example is the discrimination of biased stimulus strength in stochastic decision switches (see Figure 4.C in main text). In a stochastic decision switch, a symmetric expression state (a stable node) becomes unstable due to a

subcritical pitchfork bifurcation (and then becomes a saddle point, with the unstable direction being perpendicular to the diagonal in the phase plane). Although this bifurcation can be induced by both fast and slow signalling events, fast signals usually promote faster dynamics along the unstable direction, and this results in a faster discrimination of biased signal amplitudes. This is illustrated in Figure S6, where a decision switch operating close to the pitchfork bifurcation discriminates fast (Figure S6.A) and slow (Figure S6.B) inhibitory signals. One sees that not only discrimination performance of fast signals is steeper, but also signal noise affects this performance in a greater extent.

Role of autoregulation in symmetric progression switches

From the biological scenarios documented in the literature (see Table 1) it seems that genetic architectures of mutual activation of key transcription factors with autoregulation operate as standard (symmetric) progression switches. One possible reason inferred from our analysis is that, for moderate to strong crossinteractions, autoregulation should be exceedingly high to generate the coexistence of asymmetric expression states. What could be the role played by autoregulation in these type of circuits? Here we have investigated two possible reasons, both conferring flexibility to switch performance. One possibility is that, for a fixed cross-interaction strength where the circuit is not able to operate as a switch (being in a monostable regime), the modulation of autoactivatory loops may enable the system to work as a switch. This is demonstrated in Figure S9. For a standard switch without autoregulation (Figure S9.A) if we keep the crossinteraction-strength at $\nu = 10$, a (degradation) signal is not able to change the initial (high,high) expression state. For the same cross-interaction strength, and adding autoactivation to both circuit components, in a proper range of autoregulation strength the circuit is able to work as a switch upon the same signal (Figure S9.B). The second possibility is that autoregulation may change the combinatory of signals to which a mutual-activation switch responds. In Figure S10, we show the response of a mutual-activation architecture placed in a (high,high) expression state to independent degradation signals in the X and Y components. In the case that there is no autoregulation (Figure S10.A) the switch responds as a fuzzy OR gate (a signal affecting only one component above a given threshold induces a change of expression state). However, by adding autoactivation we need the simultaneous action of signals on both components (AND gate) to switch to the (low,low) expression state.

References

[1] Pesce M. and Schöler H. R. (2001) *Stem Cells* **19** 271-278.

[2] Suh E., Chen L., Taylor J. and Traber P. G. (1994) *Mol. Cell. Biol.* **14** 7340-51.

[3] Browning DF and Busby SJW (2004) *Nat Rev Microbiol* 2:1-9.

[4] Reményi A, Schöler HR, Wilmanns M (2004) *Nat Struct Mol Biol* 11:812-815.

[5] Kuhlman T, Zhang Z, Saier Jr. MH, Hwa T (2007) *Proc Natl Acad Sci USA* 104: 6043-6048.

[6] Istrail S, Davidson EH (2005) *Proc Natl Acad Sci USA* 102: 4954-4959.

[7] Mayo AE, Setty Y, Shavit S, Alon U (2006) *PLoS Biol* 4:555-561.

[8] Kuznetsov A, Kaern M and Kopell N (2004) *SIAM J Appl Math* 65: 392-425.

[9] Thattai M and van Oudenaarden A (2001) *Proc Natl Acad Sci USA* 98:8614-8619.

[10] Ozbudak EM, Thaitai M, Kurtser I, Grossman AD and van Oudenaarden A (2002) *Nat Rev Genet* 31:69-73.

[11] Cherry JL and Adler FR (2000) *J. Theor. Biol.* 203:117-133.

[12] Blake WJ, Kaern M, Cantor CR and Collins JJ (2003) *Nature* 422: 633-637.

[13] Gillespie DT (1977) *J Phys Chem* 81: 2340-2361

[14] Haseltine EL, Rawlings JB (2002) *J Chem Phys* 117:6959-6969.

[15] Rao CV, Arkin AP (2003) *J Chem Phys* 118:4999-5010.

[16] Guido NJ, Wang X, Adalsteinsson D, McMillen D, Hasty J, Cantor CR, Elston TC and Collins JJ (2006) *Nature* 439:856-860.

[17] Adalsteinsson D, McMillen D and Elston TC (2004) *BMC Bioinformatics* 5:24.

[18] Lu T, Volfson D, Tsimring L and Hasty J (2004) *Syst Biol* 1:121-128.

[19] Kaern M, Elston TC, Blake WJ and Collins JJ (2005) *Nat Rev Genet* 6:451-464
